# Postoperative reduction of anti‐seizure medication is associated with cognitive improvement in seizure‐free patients after anterior temporal lobectomy

**DOI:** 10.1002/epi.70395

**Published:** 2026-07-20

**Authors:** Kazushi Ukishiro, Shin‐ichiro Osawa, Maimi Ogawa, Makoto Ishida, Takafumi Kubota, Kazutoshi Konomatsu, Hidenori Endo, Kazutaka Jin

**Affiliations:** ^1^ Department of Epileptology Tohoku University Graduate School of Medicine Sendai Miyagi Japan; ^2^ Department of Neurosurgery Tohoku University Graduate School of Medicine Sendai Miyagi Japan; ^3^ Department of Neurology Tohoku University Graduate School of Medicine Sendai Miyagi Japan

**Keywords:** anterior temporal lobectomy, anti‐seizure medication, cognitive outcome, dose–response relationship, temporal lobe epilepsy

## Abstract

**Objective:**

To determine whether postoperative reduction of anti‐seizure medication (ASM) burden is associated with cognitive improvement after anterior temporal lobectomy (ATL) in seizure‐free patients.

**Methods:**

We retrospectively analyzed data for adult patients with drug‐resistant temporal lobe epilepsy who underwent unilateral ATL between March 2013 and January 2025. Analyses were restricted to patients achieving complete freedom from seizures (Engel Class I) at 1 year. ASM burden was quantified using the prescribed daily dose/defined daily dose ratio, and the reduction rate was calculated. Preoperative and 1‐year postoperative neuropsychological outcomes, including full‐scale intelligence quotient (FSIQ) and domain‐specific indices, were assessed. The effect of ASM reduction on cognitive change was evaluated using a doubly robust estimation framework integrating inverse probability of treatment weighting with multivariable outcome regression. Robustness and potential dose–response relationships were assessed via sensitivity analyses, analysis of covariance (ANCOVA), and categorical modeling.

**Results:**

Fifty‐seven seizure‐free patients were included in the primary analysis (median age at surgery, 32 years; 33 female). Greater ASM reduction was significantly associated with improved working memory (WM) (*β* = .066, *p* = .001). A positive but non‐significant trend was observed for FSIQ (*β *= .021, *p* = .062). A dose–response relationship was identified, with greater ASM reduction associated with progressively greater improvement in WM (*p* = .013). Findings were consistent across sensitivity and ANCOVA analyses.

**Significance:**

Postoperative ASM reduction was associated with domain‐specific cognitive improvement following ATL, particularly in WM. These findings support ASM tapering as a potentially modifiable factor influencing postoperative cognitive outcomes.

**Key points**

- Reduction of anti‐seizure medication (ASM) burden after anterior temporal lobectomy was associated with cognitive improvement.
- The strongest association was observed in working memory.
- A dose–response relationship was identified, with greater ASM reduction associated with greater cognitive improvement.
- Findings were consistent across multiple analytical approaches.
- Postoperative ASM reduction may be considered to optimize cognitive outcomes, with careful attention to the risk of seizure recurrence.

## 1 INTRODUCTION

Anterior temporal lobectomy (ATL) is a well‐established surgical treatment for drug‐resistant temporal lobe epilepsy (TLE), achieving seizure freedom in a substantial proportion of patients.^1^, ^2^, ^3^, ^4^, ^5^ However, concerns regarding postoperative cognitive outcomes, particularly following resections of the language‐dominant hemisphere, remain central to clinical decision‐making and patient counseling.^6^, ^7^

Although contemporary epilepsy surgery focuses on minimizing localized cognitive deficits through tissue‐sparing and function‐preserving approaches,^8^, ^9^, ^10^ the role of postoperative medical management in shaping broader cognitive trajectories has received comparatively less attention. Anti‐seizure medications (ASMs) exert dose‐dependent neurocognitive adverse effects, including sedation, reduced processing efficiency, and generalized cognitive slowing.^11^ Following successful seizure control, reduction of the ASM burden is commonly pursued in clinical practice. This raises the possibility that postoperative cognitive improvement is not solely attributable to seizure cessation; alleviation of pharmacological burden may also play a role.^12^, ^13^ Given the distributed neurocognitive effects of ASMs, certain domains such as working memory (WM) may be particularly sensitive to changes in medication load.^14^, ^15^

Prior studies examining the relationship between ASM reduction and cognitive outcomes have primarily relied on conventional regression approaches. However, these analyses are particularly vulnerable to confounding by indication, as patients with more favorable postoperative courses are more likely to undergo ASM tapering and exhibit better cognitive outcomes.^16^ Furthermore, many previous studies have included heterogeneous patient populations with varying degrees of seizure control, making it difficult to disentangle the contributions of ASM reduction from those of seizure freedom.^12^, ^13^, ^16^ As a result, the association between ASM reduction and postoperative cognitive change has not been rigorously established. To address these limitations, we designed the present study using two key methodological strategies. First, we restricted our analysis to patients who achieved complete seizure freedom (Engel Class I) at 1 year, thereby minimizing confounding related to seizure outcome. Second, we applied a doubly robust approach to inverse probability of treatment weighting with multivariable outcome regression to adjust for measured confounders, including factors related to treatment selection. Using this approach, we aimed to determine whether postoperative ASM reduction is conditionally associated with cognitive improvement following ATL.

## 2 MATERIALS AND METHODS

This study was approved by the Ethics Committee of Tohoku University Hospital (approval no. 2020–1‐083) and conducted in accordance with the strengthening the reporting of observational studies in epidemiology (STROBE) guidelines for observational studies.^17^

### 2.1 Patient selection

We conducted a retrospective review of consecutive adult patients with drug‐resistant TLE who underwent unilateral ATL at Tohoku University Hospital between March 2013 and January 2025.

The exclusion criteria were established as follows: (1) a baseline preoperative full‐scale intelligence quotient (FSIQ) <50, to ensure the validity and reliability of the neuropsychological assessments; (2) non‐native Japanese proficiency; (3) extratemporal or bilateral lesions on preoperative magnetic resonance imaging (MRI); and (4) incomplete neurocognitive follow‐up at 1 year. Baseline clinical variables, including age at surgery, sex, surgical laterality, presence of hippocampal sclerosis, and preoperative neuropsychological scores, were systematically extracted from medical records to serve as potential confounders in subsequent analyses.

### 2.2 Surgical procedure and language dominance

Language lateralization was preoperatively determined through a comprehensive evaluation incorporating functional MRI, the intracarotid amobarbital procedure (Wada test),^18^, ^19^ and the super‐selective anesthesia for functional evaluation (SAFE) test.^20^, ^21^

All patients underwent standard ATL. The posterior extent of the lateral temporal neocortical resection was tailored according to language dominance.

### 2.3 Neuropsychological assessment

Comprehensive neuropsychological assessments were administered preoperatively and at 1 year postoperatively, a time point at which surgical recovery and cognitive function are generally considered to have stabilized. Assessments included the Wechsler Adult Intelligence Scale‐III (WAIS‐III)^22^ and Wechsler Memory Scale‐Revised (WMS‐R).^23^ The WAIS‐III provides indices of verbal IQ, performance IQ, FSIQ, and domain‐specific indices including verbal comprehension, perceptual organization, WM, and processing speed (PS). The WMS‐R assesses memory function across multiple domains, including memory (verbal, visual memory, and general), attention/concentration, and delayed recall. A priori defined primary outcome measures were changes in FSIQ and the WM index, per previously established methodologies.^14^, ^24^, ^25^ FSIQ was selected to reflect global cognitive function and capture the diffuse neurocognitive burden associated with ASM use. The WM index was specifically chosen owing to its reliance on distributed frontoparietal networks, which are highly susceptible to the generalized cognitive side effects of ASMs. Cognitive change scores were calculated by subtracting the preoperative score from the postoperative score.

### 2.4 Quantification of ASM burden

The pharmacological burden of ASMs was quantified using the prescribed daily dose to defined daily dose (PDD/DDD) ratio, based on the 2026 release of the WHO anatomical therapeutic chemical (ATC) to DDD (ATC/DDD) Index, in accordance with the WHO Collaborating Centre for Drug Statistics Methodology.^26^, ^27^ The 2026 ATC/DDD Index was applied uniformly to all patients and to both pre‐ and postoperative medication data at the time of calculating PDD/DDD ratios. The total ASM load was calculated by summing the PDD/DDD ratios of all concurrently prescribed ASMs for each patient. The ASM reduction rate (%) was defined as [(preoperative ASM load − postoperative ASM load)/preoperative ASM load] × 100.

Decisions regarding ASM reduction were made based on a combination of factors, including seizure outcome, clinical stability, adverse effects, and physician judgment. ASM tapering was not protocolized and determined at the discretion of the treating physician. Neuropsychological testing was performed only preoperatively and at 1 year postoperatively; no interim cognitive assessments were available during the tapering period, and tapering decisions were based on seizure outcome, clinical stability, and patient‐reported adverse effects such as somnolence, rather than on formal cognitive test results.

### 2.5 Statistical analysis

Continuous variables are presented as median (interquartile range [IQR]), and categorical variables as number (%). Primary analyses were restricted to patients who achieved complete seizure freedom (Engel Class I) at 1 year. The association between ASM reduction and cognitive change was evaluated using a doubly robust estimation framework combining inverse probability of treatment weighting and multivariable regression. Propensity scores were estimated based on preoperative FSIQ, age at surgery, and hippocampal sclerosis. Outcome models were additionally adjusted for surgical laterality and baseline cognitive performance. Given the modest sample size, the propensity model was intentionally restricted to three, a priori clinically relevant covariates to reduce the risk of overfitting. These covariates were selected because age at surgery, preoperative FSIQ, and hippocampal sclerosis plausibly influence both postoperative cognitive outcome and clinical decisions regarding ASM reduction. Covariate balance across ASM‐reduction tertiles before and after weighting was assessed using the maximum absolute pairwise standardized mean difference, with values below 0.10 considered to indicate good balance (Table S1). FSIQ and WM were pre‐specified as the two primary outcomes; analyses of additional cognitive domains were considered exploratory and were not adjusted for multiple comparisons. The robustness of the primary WM finding to multiplicity was examined using a Bonferroni‐corrected threshold (α = .025) for the two primary outcomes.

Sensitivity analyses included comparisons across unadjusted, minimally adjusted, and fully adjusted models, as well as analysis of covariance (ANCOVA) adjusting for baseline performance. A categorical dose–response analysis was conducted by stratifying ASM reduction into tertiles, and a linear trend was assessed by modeling the ordinal groups as continuous variables. In additional sensitivity analyses, we assessed the potential influence of ASMs with known cognitive adverse effects by excluding patients who were receiving topiramate at the time of preoperative assessment.^11^ The primary analysis was then repeated in this restricted cohort using the same doubly robust estimation framework to evaluate the robustness of the observed association between ASM reduction and cognitive change.

All statistical analyses were performed using JMP Student Edition (SAS Institute Inc., Cary, NC, USA). Statistical significance was defined as a two‐sided *p*‐value of <.05.

## 3 RESULTS

### 3.1 Patient selection and flow

During the study period, 79 patients underwent ATL (Figure 1). Of these, 67 met the inclusion criteria, and 12 were excluded for the following reasons: intellectual disability (*n* = 4), loss to follow‐up (*n* = 3), inability to complete postoperative neuropsychological assessment owing to the coronavirus disease 2019 (COVID‐19) pandemic (*n* = 3), and non‐native Japanese proficiency (*n* = 2). Among the 67 eligible patients, 57 achieved complete seizure freedom (Engel Class I) at 1 year and were included in the primary analysis to eliminate seizure‐related confounding.

**FIGURE 1:**
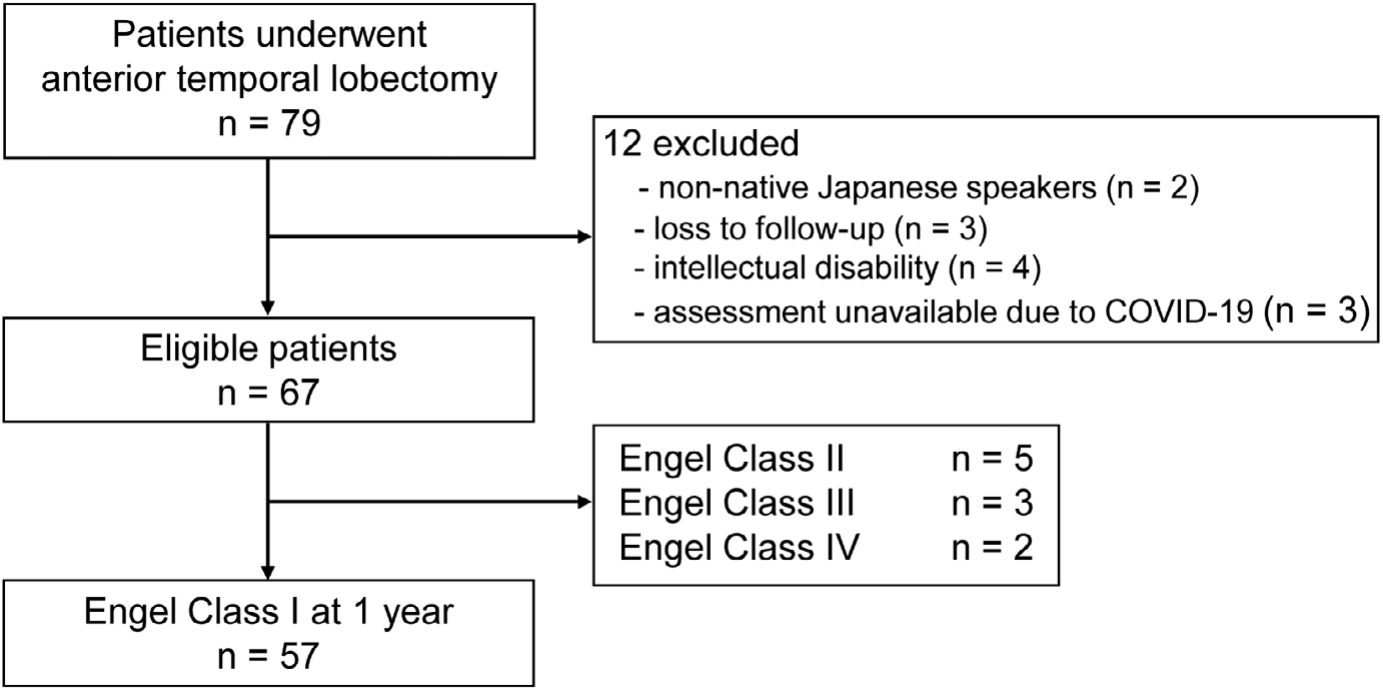
Flow diagram of patient selection. Of 79 patients who underwent anterior temporal lobectomy (ATL), 67 met the inclusion criteria after predefined exclusions. Among these, 57 patients achieved complete seizure freedom (Engel Class I) at 1 year and were included in the primary analysis.

### 3.2 Patient characteristics

This seizure‐free cohort comprised 33 female patients, and 54 patients were right‐handed. Baseline characteristics of the study population are summarized in Table 1. Language lateralization was determined using functional MRI or SAFE testing when available. For 11 right‐handed patients without functional data, left hemispheric language dominance was assumed based on handedness and concordant neuropsychological profiles. Thirty‐one patients underwent ATL in the language‐dominant hemisphere. Hippocampal sclerosis was pathologically confirmed in 39 patients. The median ages at seizure onset and surgery were 16 (IQR, 12–25) and 32 (24–43.3) years, respectively. The median ASM reduction rate was 25% (0%–53.8%). The median number of ASMs decreased from 3 (2–3) preoperatively to 2 (2–3) at 1 year postoperatively. The median FSIQ increased from 84 (74–99.3) preoperatively to 86 (74–100.5) postoperatively, whereas the median WM index improved from 85 (74–94) to 88 (79–105.5).

**TABLE 1:** Patient characteristics.

Characteristics	Value (*n* = 57)
Female sex	33 (57.9)
Right‐handed	54 (94.7)
Dominant‐hemisphere ATL	31 (54.4)
Left ATL	29 (50.9)
Hippocampal sclerosis	39 (68.4)
Age at seizure onset, years	16 (12–25)
Age at surgery, years	32 (24–43.3)
Preoperative number of ASMs	3 (2–3)
Postoperative number of ASMs	2 (2–3)
ASM reduction rate, %	25 (0–53.8)
Topiramate use (preoperative)	6 (10.5)
Preoperative FSIQ	84 (74–99.3)
Postoperative FSIQ	86 (74–100.5)
Preoperative WM	85 (74–94)
Postoperative WM	88 (79–105.5)

*Note*: Baseline demographic and clinical characteristics of patients with drug‐resistant temporal lobe epilepsy undergoing anterior temporal lobectomy who were included in the primary analysis. Continuous variables are presented as median (interquartile range), and categorical variables are presented as number (percentage).

Abbreviations: ASM, anti‐seizure medication; ATL, anterior temporal lobectomy; FSIQ, full‐scale intelligence quotient; WM, working memory.

### 3.3 Primary analysis: Association between ASM reduction and cognitive change

Using the doubly robust estimation framework (Figure 2), ASM reduction was associated with favorable cognitive changes. The strongest association was observed for WM, with greater ASM reduction associated with larger improvements in WM (*β* = .066, 95% confidence interval [CI]: .027–0.105, *p* = .001). Weighting reduced the maximum pairwise standardized mean difference among propensity‐model covariates from 0.51 to 0.13. Post‐weighting values were .06 for age at surgery, 0.13 for preoperative FSIQ, and .01 for hippocampal sclerosis, indicating substantially improved balance, although minor residual imbalance remained for preoperative FSIQ. Therefore, baseline cognitive performance was additionally included in the outcome‐regression component of the doubly robust model.

**FIGURE 2:**
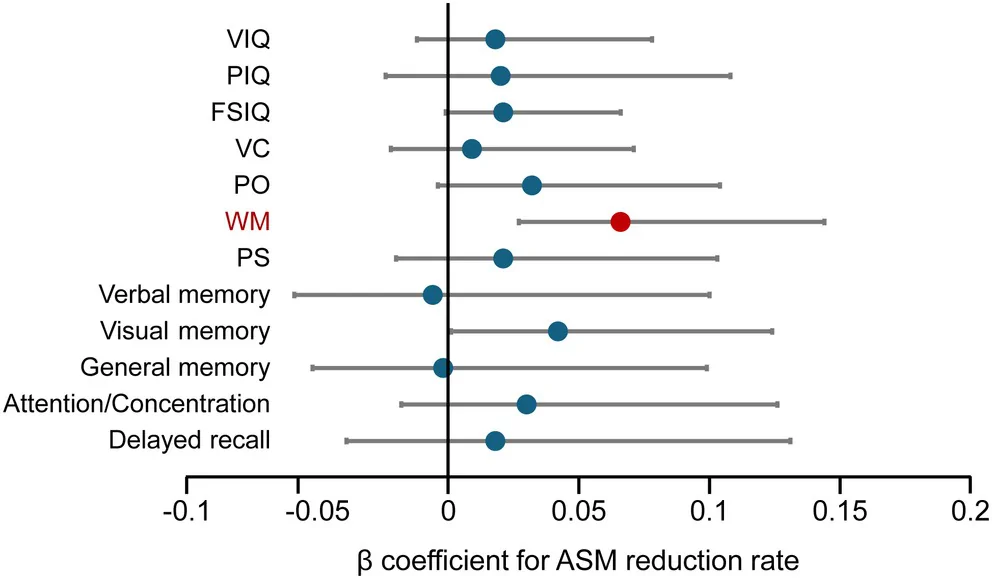
Association between anti‐seizure medication (ASM) reduction and changes in cognitive domains. Forest plot showing the estimated effect of the ASM reduction rate on changes in cognitive performance across domains. Points represent regression coefficients (*β*), and horizontal lines indicate 95% confidence intervals derived from fully adjusted doubly robust models. Positive values indicate greater cognitive improvement with greater ASM reduction. The vertical line at zero indicates no association. Working memory showed the strongest association, and most domains demonstrated positive point estimates.

A positive but non‐significant trend was observed for FSIQ (*β* = .021, 95% CI: −.001 to .044, *p* = .062). In exploratory analyses, visual memory showed a modest nominal association, whereas other domains showed no significant associations (Table S2).

### 3.4 Sensitivity and robustness analyses

The association between ASM reduction and WM improvement was consistent across all models (Table 2), with similar effect estimates in unadjusted, minimally adjusted, and fully adjusted analyses (*β* = .066). In ANCOVA models, ASM reduction remained significantly associated with postoperative WM (*β* = .066, 95% CI: .027–.105, *p* = .001), and baseline WM was also strongly associated with postoperative WM (*β* = .958, *p* < .001). Results remained consistent after exclusion of patients receiving topiramate (*β* = .058, 95% CI: .015–0.101, *p* = .01; Table S3). The WM association remained significant under Bonferroni correction for the two pre‐specified primary outcomes (α = .025).

**TABLE 2:** Association between ASM reduction and WM outcomes.

Analysis	Outcome	*β*	95% CI	*p*‐value
Unadjusted	WM change	.066	.028–.105	.001
Minimally adjusted	WM change	.066	.028–0.104	.001
Fully adjusted (doubly robust)	WM change	.066	.027–0.105	.001
ANCOVA	Postoperative WM	.066	.027–0.105	.001

*Note*: Regression coefficients (*β*) represent the estimated effect of the ASM reduction rate on the change in working memory (WM) or postoperative WM score. The fully adjusted model was based on a doubly robust estimation framework combining inverse probability of treatment weighting and multivariable regression. The ANCOVA model used postoperative WM score as the dependent variable, adjusting for baseline WM and other covariates.

Abbreviations: ANCOVA, analysis of covariance; ASM, anti‐seizure medication; CI, confidence interval; WM, working memory.

### 3.5 Dose–response relationship

A linear trend analysis confirmed a significant dose–response relationship (Figure 3, *p* = .013). This categorical analysis demonstrated a clear dose‐dependent pattern: patients in the lowest reduction group showed minimal change or a slight decline in WM performance (mean change = −1.1), whereas those in the middle and highest reduction groups exhibited progressively greater improvements (mean changes = 8.2 and 10.9, respectively).

**FIGURE 3:**
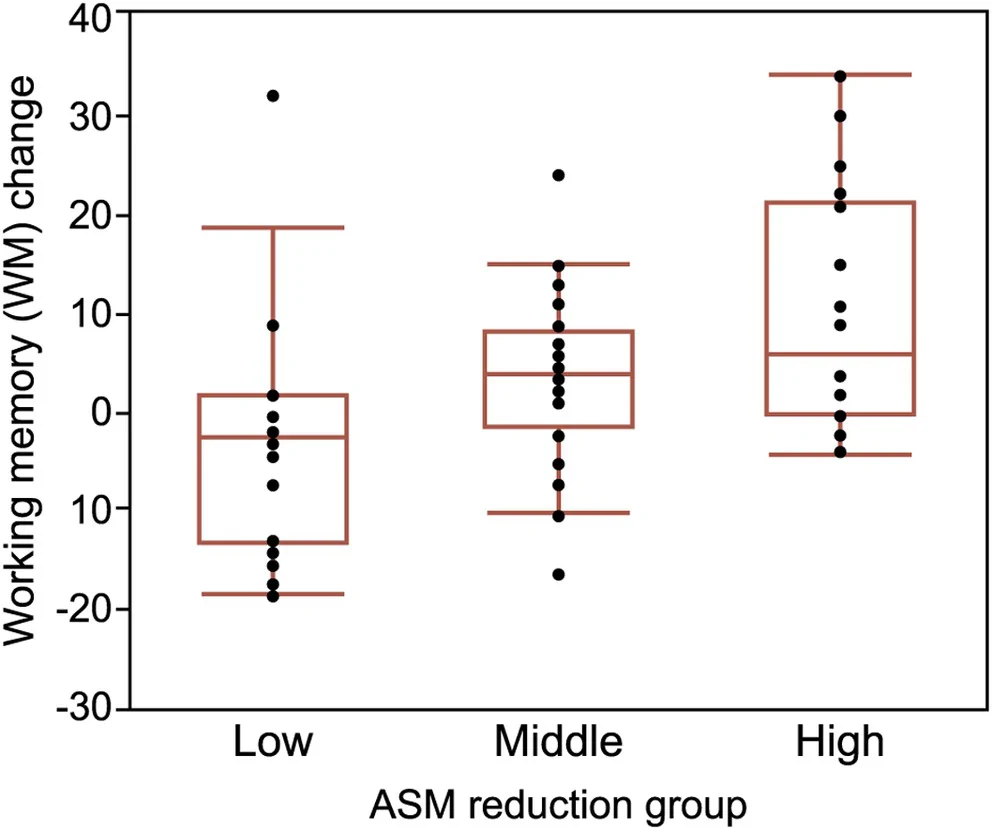
Dose–response relationship between anti‐seizure medication (ASM) reduction and working memory (WM) improvement. Patients were stratified into three groups according to the degree of postoperative ASM reduction. Changes in WM are shown for each group. Greater ASM reduction was associated with progressively greater improvement in WM.

## 4 DISCUSSION

The current findings revealed that greater postoperative reduction in ASM burden was associated with cognitive improvement following ATL, with the most pronounced effect observed in WM. This association was consistent across multiple analytical approaches, including doubly robust estimation and analysis of covariance, and a dose–response relationship was identified, with greater ASM reduction associated with greater cognitive gains. By restricting our analysis to a seizure‐free cohort (Engel Class I) and applying a doubly robust estimation framework, we aimed to minimize confounding by seizure outcome and indication. The consistency of findings across different model specifications supports the robustness of the observed association and suggests that the baseline differences in patient characteristics do not solely explain the relationship between ASM reduction and cognitive improvement.

### 4.1 Domain‐specific sensitivity to pharmacological burden

The domain‐specific patterns of cognitive change observed in this study provide insight into the potential neurobiological mechanisms underlying postoperative recovery. WM appeared particularly sensitive to ASM reduction, whereas cognitive functions more directly related to mesial temporal lobe structures, such as verbal memory, showed more limited responsiveness. This pattern is anatomically plausible. WM depends on distributed frontoparietal networks that are largely preserved following ATL but may be susceptible to the diffuse cognitive effects of ASMs.^11^ Reduction of pharmacological burden may therefore facilitate functional improvement within these intact networks. In contrast, cognitive functions relying on mesial temporal structures are directly affected by surgical resection, and structural loss may limit the extent to which medication reduction translates into measurable cognitive gains.^3^, ^28^, ^29^, ^30^, ^31^

A modest association was also observed for visual memory. However, this finding should be interpreted with caution, given the multiple comparisons and the relatively small effect size. Further studies are needed to clarify whether this reflects a true domain‐specific effect or a chance finding.

### 4.2 Dose–response relationship and robustness

The categorical analysis demonstrated a clear dose–response relationship, with progressively greater WM improvements at higher levels of ASM reduction. Patients with minimal reductions showed little to no improvement, whereas those with greater reductions exhibited more substantial gains. Although a dose–response relationship was evident at the group level, the distributions overlapped substantially across tertiles (Figure 3), and many patients with large ASM reductions showed little cognitive improvement. ASM reduction, therefore, accounts for only part of the variability in postoperative WM change and should be considered one of several contributing factors. This pattern was consistent across multiple analytical approaches, including unadjusted, minimally adjusted, fully adjusted, and ANCOVA models. The convergence of these findings supports the interpretation that ASM reduction may contribute to cognitive improvement, although residual confounding cannot be entirely excluded. Because formal cognitive data were not available to clinicians during the tapering period, reverse causation—whereby cognitive improvement prompted subsequent ASM reduction—is less likely to explain the observed association. Of note, this association was not driven by specific agents with known cognitive adverse effects, as exclusion of patients receiving topiramate yielded consistent results. This supports the interpretation that the observed effect reflects a broader reduction in pharmacological burden rather than drug‐specific effects. The near‐identical effect estimates across the unadjusted, minimally adjusted, fully adjusted, and ANCOVA models may appear surprising. This convergence indicates that adjustment for the covariates included in our models did not materially alter the exposure coefficient; thus, confounding by these variables for the WM outcome was limited. In addition, the similarity between the ANCOVA and change‐score estimates was likely related to the near‐unity baseline working‐memory coefficient in the ANCOVA model (*β* = .958), because adjustment for a baseline value with a coefficient close to 1 can approximate a change‐score parameterization. This stability does not exclude residual confounding from unmeasured factors.

### 4.3 Clinical implications

Clinicians often exercise caution when reducing ASMs in the early postoperative period due to concerns regarding seizure recurrence. However, previous studies suggest that, for appropriately selected patients, earlier initiation of ASM withdrawal may not adversely affect long‐term seizure outcomes.^16^, ^32^, ^33^ In the present cohort, ASM reduction was generally initiated during the first postoperative year in seizure‐free patients. Although the timing of withdrawal was not standardized, the present findings suggest that a reduction in ASM burden during this period may be associated with cognitive benefit, particularly in WM. Taken together, these results indicate that postoperative ASM management may be considered, not only in terms of seizure control but also as a potentially modifiable factor influencing cognitive outcomes. Incorporating cognitive function into postoperative decision‐making may help guide a more balanced and individualized approach to ASM tapering while maintaining careful attention to the risk of seizure recurrence. Given the substantial individual variability observed, ASM reduction should be considered one of several modifiable contributors to cognitive outcome rather than a guaranteed benefit, and tapering decisions should remain individualized.

### 4.4 Strengths

This study has several strengths. The restriction to a seizure‐free cohort minimized confounding by seizure outcome. The use of a doubly robust estimation framework enhanced our causal inference. Furthermore, the consistency of findings across multiple analytical approaches supports the robustness of the results.

### 4.5 Limitations

Some limitations warrant mention. First, this was a retrospective, single‐center study conducted in a Japanese cohort, which may limit the generalizability of our findings. Neuropsychological instruments, cultural and linguistic factors, and ASM prescribing practices may differ across international epilepsy surgery centers. Second, ASM tapering was not protocolized and was determined at the discretion of the treating physician. Patients undergoing greater ASM reduction may have had more favorable postoperative clinical trajectories, potentially introducing residual confounding by indication. Although we applied a doubly robust estimation framework to adjust for measured covariates, residual confounding from unmeasured factors—such as educational attainment, mood or psychiatric symptoms, duration of epilepsy, baseline ASM class, adverse effects, medication adherence, patient preference, and physician judgment regarding seizure recurrence risk—cannot be excluded. Because the decision to reduce ASMs was based on each patient's postoperative course, it may have incorporated information not fully captured by measured covariates. Third, the ASM burden was quantified using a composite metric (PDD/DDD ratio), which does not account for drug‐specific cognitive effects. Certain ASMs, such as topiramate, are known to have disproportionately strong cognitive adverse effects. Therefore, the observed associations may not fully capture the heterogeneity of cognitive impact across individual ASM agents. Fourth, the neuropsychological assessment used the WAIS‐III rather than a more recent edition. We selected this instrument to maintain consistency across the entire study period and to permit valid intra‐subject comparison; nevertheless, the older normative data represent a limitation. In addition, we did not apply reliable change indices or formal practice‐effect corrections. Practice effects may have contributed to postoperative score changes and can vary across individuals according to baseline ability, age, clinical status, and test familiarity; however, because all patients underwent the same testing schedule, a uniform practice effect would be expected to affect the reduction groups similarly and is unlikely to fully explain the observed dose–response relationship. Finally, neuropsychological assessment at 1 year postoperatively may reflect cognitive changes occurring during the tapering phase; longer‐term follow‐up is needed to determine whether these effects persist after complete ASM withdrawal.

## 5 CONCLUSION

Postoperative ASM reduction was associated with cognitive improvement following ATL, with the most robust effect observed in WM. The presence of a dose–response relationship and consistency across analytical approaches supports the potential of ASM reduction as a modifiable factor influencing postoperative cognitive outcomes. Further prospective, multicenter studies are needed to confirm these findings and establish optimal strategies for postoperative ASM management.

## AUTHOR CONTRIBUTIONS

**Kazushi Ukishiro**, **Shin‐ichiro Osawa**, and **Maimi Ogawa**: Drafting and critical revision of the manuscript; study conception and design. **Makoto Ishida**: Analysis or interpretation of data. **Takafumi Kubota and Kazutoshi Konomatsu**: Major role in data acquisition. **Hidenori Endo**: Study conception and design. **Kazutaka Jin**: Major role in data acquisition; study conception and design. All the authors have read and approved the final version of the manuscript.

## FUNDING INFORMATION

This study received no specific funding.

## CONFLICT OF INTEREST STATEMENT

The authors declare no conflict of interest.

## ETHICS PUBLICATION STATEMENT

This study was approved by the Ethics Committee of Tohoku University Hospital (approval no. 2020–1‐083). We confirm that we have read the Journal's position on issues involved in ethical publication and affirm that this report is consistent with those guidelines.

## PATIENT CONSENT STATEMENT

Given the retrospective design of the study, the requirement for written informed consent was waived, and an opt‐out consent procedure was used.

## Supporting information


**Table S1.** Covariate balance across anti‐seizure medication (ASM)–reduction tertiles before and after stabilized inverse probability of treatment weighting.
**Table S2**. Association between anti‐seizure medication (ASM) reduction and cognitive domains.
**Table S3**. Sensitivity analysis excluding patients receiving topiramate.

## Data Availability

Data are not publicly available because of privacy and ethical restrictions but will be available from the corresponding author upon reasonable request.
