# Supplementary material for: Postoperative reduction of anti‐seizure medication is associated with cognitive improvement in seizure‐free patients after anterior temporal lobectomy
Source: Epilepsia. 2026 Jul 20;67(10):5255–63. doi: 10.1002/epi.70395 (PMC13675761; doi:10.1002/epi.70395)
Supplement: Supplementary file 1 [file EPI-67-5255-s001.docx]

**Supplementary Table S1.** **Covariate balance across ASM-reduction tertiles before and after stabilized inverse probability of treatment weighting**

| **Covariate** | **Included in propensity model** | **Maximum \|SMD\|**  **before IPTW** | **Maximum \|SMD\|  after IPTW** |
| --- | --- | --- | --- |
| Age at surgery | Yes | 0.30 | 0.06 |
| Preoperative FSIQ | Yes | 0.51 | 0.13 |
| Hippocampal sclerosis | Yes | 0.49 | 0.01 |
| Preoperative WM | No, included in  outcome model | 0.41 | 0.25 |
| Surgical laterality,  dominant hemisphere | No, included in outcome model | 0.16 | 0.11 |

Values indicate the maximum absolute pairwise standardized mean difference across the three ASM-reduction tertiles for each covariate, before and after stabilized inverse probability of treatment weighting. Among covariates included in the propensity model, weighting reduced the largest standardized difference from 0.51 to 0.13, indicating substantially improved balance, although minor residual imbalance remained for preoperative FSIQ. For completeness, covariates included in the outcome model but not in the propensity model are also shown. Residual differences in these variables were adjusted for in the outcome-regression component of the doubly robust estimator. Hippocampal sclerosis was coded as present = 1 and absent = 0. Surgical laterality was coded as language-dominant-hemisphere ATL = 1 and nondominant-hemisphere ATL = 0.

Abbreviations: ASM, antiseizure medication; ATL, anterior temporal lobectomy; FSIQ, full-scale intelligence quotient; IPTW, inverse probability of treatment weighting; SMD, standardized mean difference; WM, working memory

**Supplementary Table S2. Association between ASM reduction and cognitive domains**

| **Outcome** | **β** | **95% CI** | **p-value** |
| --- | --- | --- | --- |
| VIQ | 0.018 | -0.012 to 0.048 | 0.239 |
| PIQ | 0.020 | -0.024 to 0.064 | 0.367 |
| FSIQ | 0.021 | -0.001 to 0.044 | 0.062 |
| VC | 0.009 | -0.022 to 0.040 | 0.569 |
| PO | 0.032 | -0.004 to 0.068 | 0.080 |
| WM | 0.066 | 0.027 to 0.105 | 0.001 |
| PS | 0.021 | -0.020 to 0.062 | 0.310 |
| Verbal  memory | -0.006 | -0.059 to 0.047 | 0.815 |
| Visual  memory | 0.042 | 0.001 to 0.083 | 0.043 |
| General  memory | -0.002 | -0.052 to 0.049 | 0.952 |
| Attention/  concentration | 0.030 | -0.018 to 0.078 | 0.212 |
| Delayed  recall | 0.018 | -0.039 to 0.074 | 0.527 |

Regression coefficients (β) represent the estimated effect of ASM reduction rate on changes in cognitive test scores across domains, derived from the fully adjusted doubly robust model. Positive values indicate greater cognitive improvement with greater ASM reduction. ASM, antiseizure medication; CI, confidence interval; FSIQ, full-scale intelligence quotient; PIQ, performance intelligence quotient; PO, perceptual organization; PS, processing speed; VC, verbal comprehension; VIQ, verbal intelligence quotient; WM, working memory.

**Supplementary Table S3. Sensitivity analysis excluding patients receiving topiramate**

| **Analysis** | **Outcome** | **β** | **95% CI** | **p-value** |
| --- | --- | --- | --- | --- |
| Fully adjusted (doubly robust, excluding TPM) | WM change | 0.058 | 0.015–0.101 | 0.01 |

Effect estimates for the association between ASM reduction and working memory after exclusion of patients receiving topiramate. Analyses were performed using the same doubly robust estimation framework as in the primary analysis. TPM, topiramate; WM, working memory
